# Discovery of Protein-Derived Candidate Anticancer Peptides from Toad Poison (ChanSu) Using an Integrated Proteomics and Bioinformatics-Guided Strategy

**DOI:** 10.3390/toxins18080326

**Published:** 2026-07-27

**Authors:** Juan Chen, Bing Wang, Yingying Xie, Fei Xue, Li Shi, Yang Jiao, Yongqiang Lin

**Affiliations:** 1 Institute of Pharmacy, Shandong University of Tradional Chinese Medicine, Jinan 250355, China; 2 State Key Laboratory of Drug Regulatory Science, National Institutes for Food and Drug Control, Beijing 100061, China; 3 NMPA Key Shandong Engineering Laboratory for Standard Innovation and Quality Evaluation of TCM, Shandong Engineering Research Center for Generic Technologies of Traditional Chinese Medicine Formula Granules, Laboratory for Quality Evaluation of Gelatin Products, Shandong Institute for Food and Drug Control, Jinan 250101, China

**Keywords:** proteomics, anticancer peptides, toad poison, peptide, peptidomics

## Abstract

Toad poison (ChanSu), a traditional animal-derived medicine, has long been used in East Asian medical systems for the treatment of inflammatory conditions and tumor-related disorders. While bufadienolides have been extensively investigated as its major bioactive constituents, the contribution of peptide components to its antitumor effects remains largely unexplored. Here, we identified protein-derived candidate antiproliferative peptides from toad poison using an integrated proteomics and bioinformatics-guided strategy. Proteomic analysis identified 135 proteins, from which 2117 peptide sequences were generated via in silico digestion with trypsin and pepsin. Subsequent multi-step screening using PeptideRanker, AntiCP, iACP, and ACPred yielded twelve candidate peptides with predicted anticancer activity. Network pharmacology analysis suggested their potential involvement in cancer-related targets and pathways. ADMET (Absorption, Distribution, Metabolism, Excretion, and Toxicity) evaluation was subsequently used as a complementary assessment of drug-like and safety-related properties, further prioritizing four peptides for experimental validation, while molecular docking supported their interactions with key lung cancer-associated targets. In vitro assays demonstrated that three of the four prioritized peptides (WEAWN, NSQWG, and ACGVIGICQ) exhibited initial antiproliferative activity against human lung cancer A549 cells, though these findings should be interpreted with caution given the absence of a positive control in the MTT assay. This study provides preliminary evidence suggesting that protein-derived peptide candidates from toad poison may possess antiproliferative potential, representing an early systematic exploration of its previously unexplored peptidome as a source of candidate antiproliferative peptides warranting further pharmacological investigation. The integrated strategy presented here offers an efficient approach for the discovery of bioactive peptides from animal-derived traditional medicines.

## 1. Introduction

Lung cancer remains one of the leading causes of cancer-related mortality worldwide and ranks among the most prevalent malignancies affecting both men and women [1,2]. Despite advances in surgical intervention, radiotherapy, chemotherapy, and targeted therapy, therapeutic outcomes remain unsatisfactory for many patients, largely due to drug resistance and adverse effects [3,4]. Therefore, the exploration of novel therapeutic agents with improved efficacy and safety profiles remains an urgent priority.

Traditional Chinese medicines derived from animals represent a unique category of natural therapeutics rich in proteins and peptides. These bioactive macromolecules are widely distributed in animal organs, secretions, and physiological or pathological products, and are increasingly recognized as important contributors to the therapeutic effects of animal-based traditional medicines. Proteins and peptides derived from animal sources exhibit diverse biological activities, including antiangiogenic, antibacterial, antifungal, and anticancer effects [5,6,7].

Toad poison (ChanSu), the dried secretion of the parotid glands of Bufo bufo gargarizans Cantor or Bufo melanostictus Schneider [8], has been used for centuries in traditional Chinese medicine for the treatment of various ailments. It is widely utilized in several Asian countries, including China, Korea, and Japan [9]. According to traditional medical records, toad poison is primarily used for detoxification and analgesia [10,11]. The Chinese Pharmacopoeia (2020 edition) further documents its pharmacological activities, including detoxifying, analgesic, and cardiotonic effects, and its clinical applications in conditions such as ulcers, sore throat, sunstroke, fever, abdominal pain, vomiting, and diarrhea [8]. Chemically, toad poison contains a variety of bioactive constituents, including amino acids, alkaloids, bufogenins, and bufotoxins [12,13,14,15,16], which contribute to its broad pharmacological profile and extensive clinical use.

To date, most studies on the pharmacologically active constituents of toad poison have predominantly focused on small-molecule compounds, particularly bufadienolides. In contrast, the potential contribution of protein- and peptide-derived components to its therapeutic effects, especially its traditionally recognized antitumor activity, has received comparatively limited attention. Considering the well-documented biological activities of amphibian-derived peptides, it is plausible that peptide components may also contribute to the pharmacological basis of toad poison.

Bioactive peptides have emerged as promising therapeutic candidates with potential applications in disease prevention and clinical treatment [17]. In the field of oncology, increasing evidence suggests that naturally derived peptides can exert potent anticancer effects through diverse mechanisms. For instance, melittin, a cationic peptide isolated from bee poison, has demonstrated significant anticancer activity by disrupting cellular membranes, inducing mitochondrial dysfunction, and promoting apoptosis [18]. Similarly, lactoferricin B, a peptide fragment derived from bovine lactoferrin, exhibits pronounced anticancer activity primarily through direct membrane disruption and induction of apoptotic pathways [19]. These findings highlight the therapeutic potential of natural peptides as alternative anticancer agents.

Given the vast diversity of naturally occurring peptides and the labor-intensive nature of conventional bioactivity screening, efficient strategies for identifying candidate anticancer peptides are urgently needed. Virtual screening approaches integrating bioinformatics analysis, molecular docking, and related computational techniques have been increasingly applied for the prediction of bioactive peptides [20,21,22]. Structure-based databases and physicochemical property analyses enable the identification of candidate sequences with potential biological activities through residue interaction patterns and functional prediction models [23,24,25,26]. The integration of proteomics with virtual screening provides a powerful strategy for systematically mining peptide components from complex natural matrices, thereby improving screening efficiency and success rates [27].

**Figure 1:**
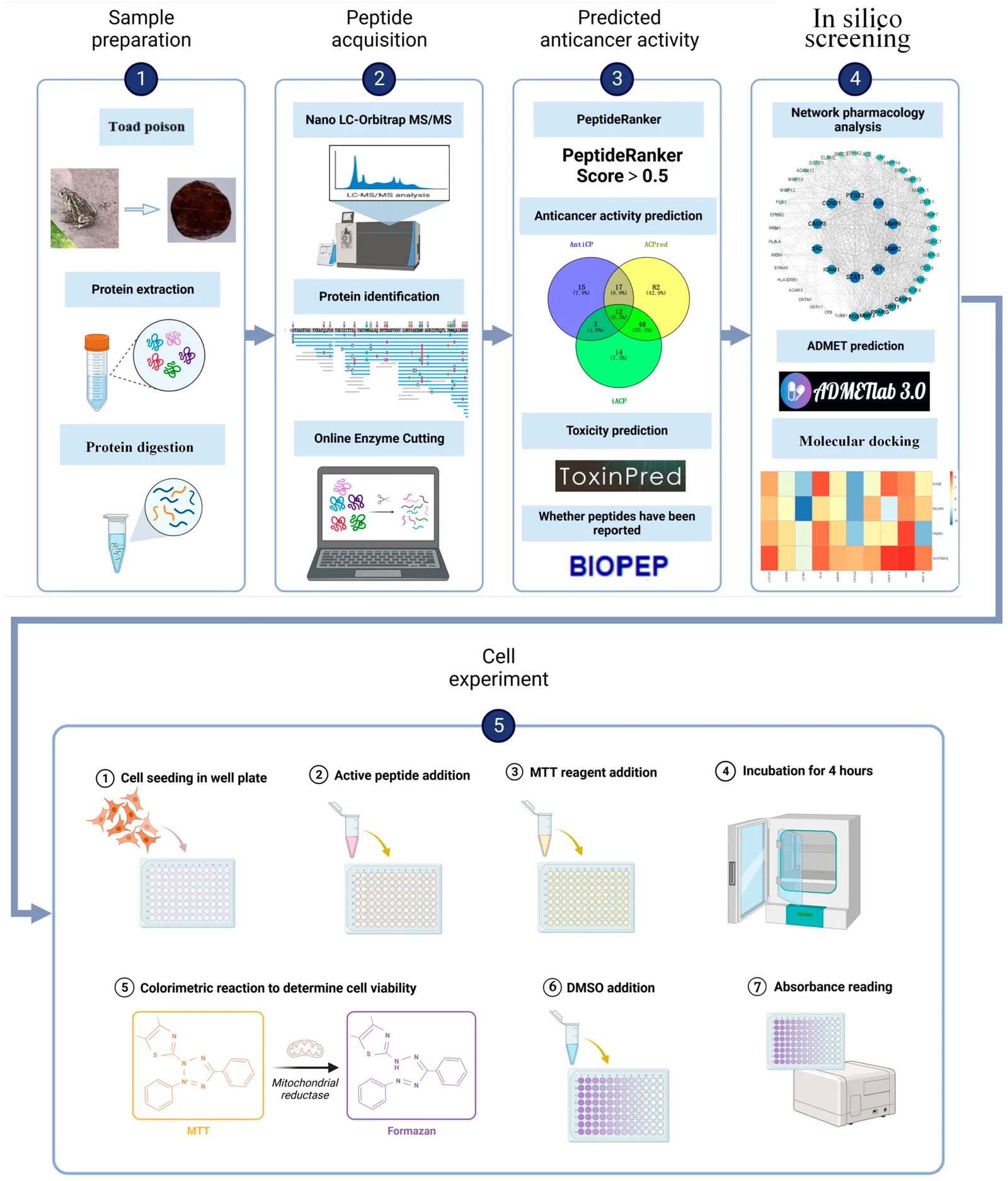
Analytical scheme for discovering active peptides in toad poison and the in vitro lung cancer inhibition assay analysis.

In the present study, a proteomics-based strategy using Nano LC–Orbitrap MS was employed to comprehensively identify protein components in toad poison. Candidate peptide sequences were generated through in silico enzymatic digestion and subjected to multi-step bioinformatics-based screening to identify potential anticancer peptides. Network pharmacology analysis was conducted to explore potential cancer-related targets and pathways. ADMET profiling and molecular docking were further performed to evaluate pharmacokinetic properties and target-binding potential. Selected peptides were subsequently evaluated in preliminary in vitro antiproliferative assays to provide initial experimental support for the computational predictions. This integrated workflow aims to systematically explore peptide components in toad poison and to provide new insights into the macromolecular pharmacological basis underlying its traditional antitumor application. Figure 1 presents a schematic overview of the experimental design.

## 2. Results

### 2.1. Determination of Protein Content

Protein concentration was determined using the Bradford method. The standard curve yielded the regression equation Y = 2.3764X + 0.4184 (R^2^ = 0.9952), where Y is the absorbance at 595 nm and X is the protein concentration (mg/mL). The protein contents of the 14 toad poison batches are summarized in Table 1.

### 2.2. Results of Nano LC-MS/MS Data Analysis

**Figure 2:**
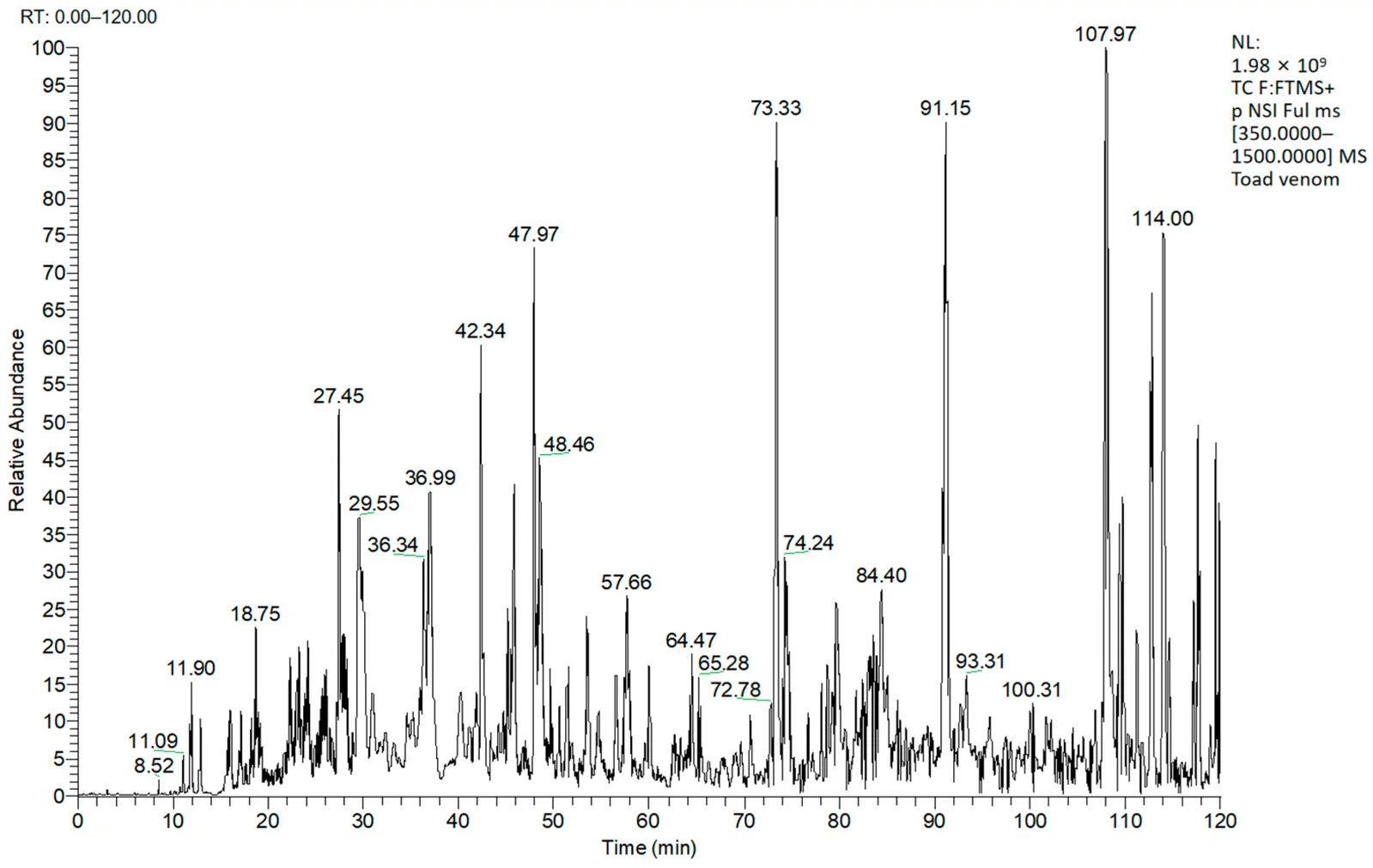
Total Ion Current diagram. Representative total ion current (TIC) chromatogram of the toad poison sample TV-SD-1.

The Total Ion Current (TIC) is illustrated in Figure 2. The Peaks software search identified 1302 protein sequences from the NCBI Bufo bufo gargarizans Cantor database, of which 135 protein sequences were selected for subsequent analysis based on their content and credibility.

### 2.3. Online Enzyme Cutting

The initial set of 135 protein sequences was subjected to in silico digestion using the ExPASy PeptideCutter tool (https://web.expasy.org/peptide_cutter/, accessed on 15 October 2025) with both pepsin and trypsin. To maximize peptide sequence coverage, peptide fragments generated by the two enzymes were combined, and duplicate sequences were removed. This process yielded 2117 unique peptide sequences ranging from 5 to 40 amino acids in length for subsequent screening.

### 2.4. Results of Prediction of the Anticancer Activity of Toad Poison Peptides

**Figure 3:**
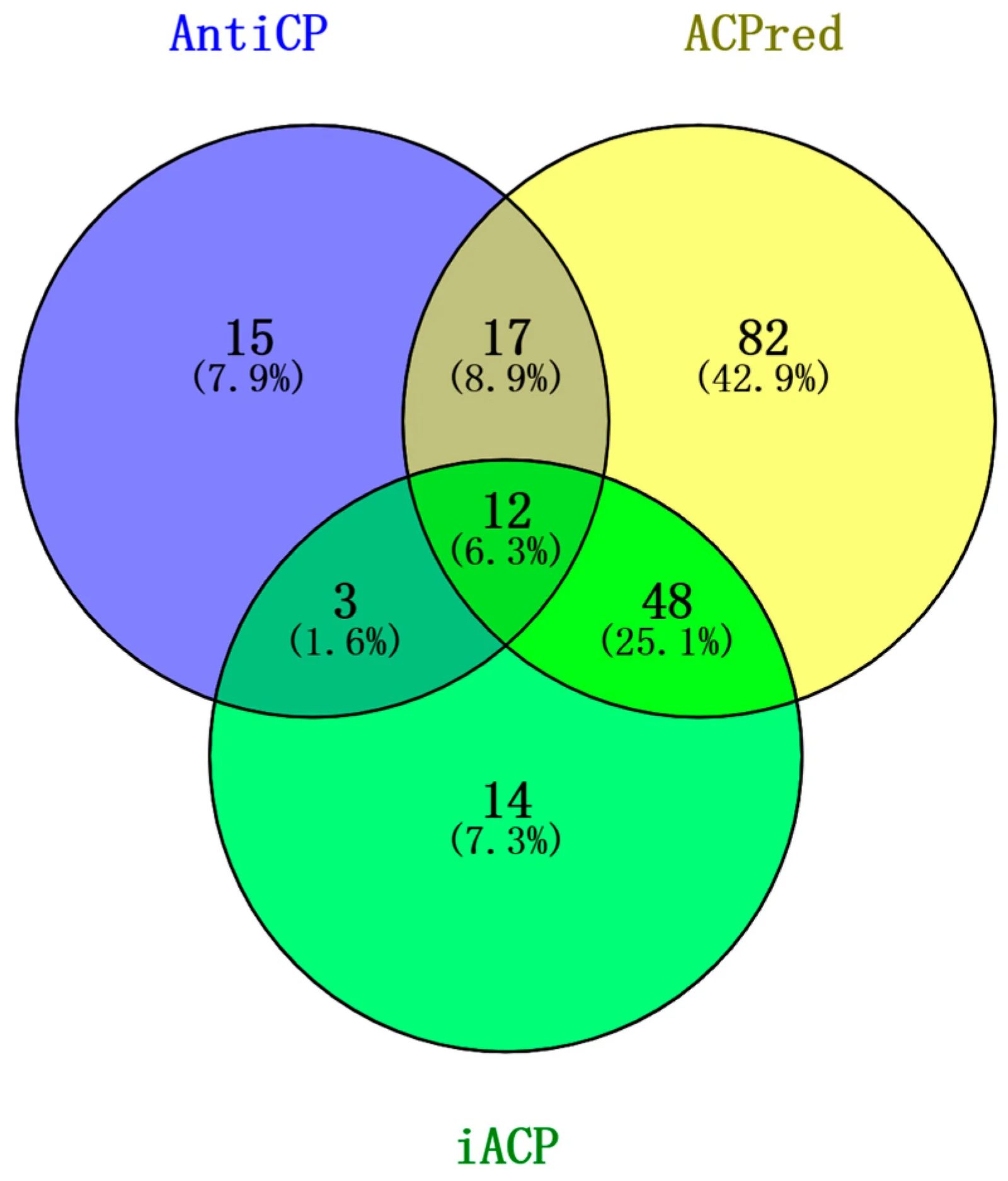
Venn diagram of the number of predicted anticancer peptides from AntiCP, ACPred, Iacp.

PeptideRanker evaluated 2117 peptide sequences for bioactivity, identifying 249 peptides with scores exceeding 0.5 and categorizing them as active. Among these, 47 peptides were predicted to possess antitumor activity by AntiCP, 159 by ACPred, and 76 by iACP. The collective prediction from these tools pinpointed 12 peptides exhibiting antitumor potential (Figure 3). Toxinpred was employed to evaluate the toxicity of these 12 peptides, indicating their non-toxic nature. The sequences of these 12 peptides are as follows: FGTQF, RPPGR, WEAWN, FSPDGH, NSQWG, FSTHL, AEWGR, NEYWR, RPFER, STWII, ACGVIGICQ, and IAWTR. Subsequently, the amino acid sequences of these 12 peptides were queried in the BIOPEP database for known functions, but no matching functions were identified. The PeptideRanker scores for these 12 peptide sequences are presented in Table 2.

### 2.5. Network Pharmacology Analysis

#### 2.5.1. Target Prediction of Active Peptide and Lung Cancer

**Figure 4:**
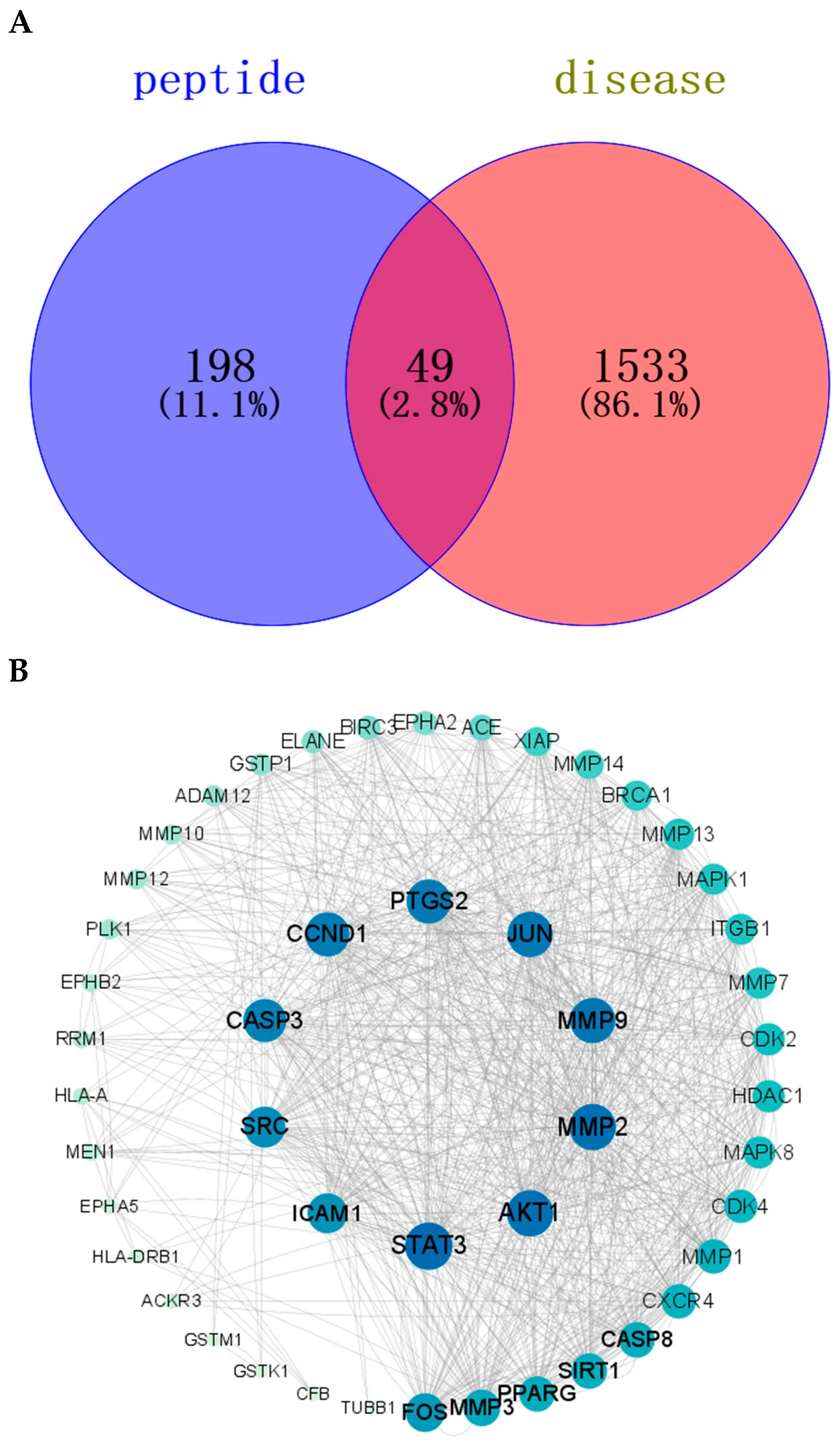
(**A**) Venn diagram of the target of 12 peptide sequences and the target of lung cancer. (**B**) The PPI network of 12 peptide sequences and lung cancer targets. Nodes represent proteins (The colors from light green to dark represent the degree of binding between proteins; larger nodes indicate higher degree values, reflecting a greater likelihood of being a core target). Edge represents protein-protein association.

The SwissTargetPrediction website forecasted 247 targets for 12 active peptide sequences. Simultaneously, 1582 targets were uncovered through a search across different databases (OMIM, DrugBank, Therapeutic Target Database, GeneCards, and DisGeNET) for targets associated with lung cancer. A Venn diagram (Figure 4A) depicted the intersection between active peptide ingredients and disease targets, highlighting the identification of 49 potential therapeutic targets for lung cancer from the 12 peptides.

It should be noted that, to the best of our knowledge, no currently available web-based platform provides proteome-wide target prediction for short peptides with functionality comparable to that of SwissTargetPrediction for small molecules. Existing peptide–protein interaction prediction methods are generally designed to evaluate interactions between a peptide and a pre-specified target protein, rather than to perform unbiased proteome-wide target identification. In the absence of a peptide-specific platform with comparable functionality, SwissTargetPrediction was employed as an exploratory target prediction tool, with the explicit acknowledgment that its applicability to peptide ligands may be limited. Accordingly, the predicted targets and all downstream network pharmacology analyses, including PPI, GO, and KEGG enrichment analyses, should be interpreted as exploratory and hypothesis-generating rather than as definitive mechanistic evidence.

#### 2.5.2. PPI Network Construction and Analysis

The Protein–Protein Interaction (PPI) network was formulated to visually represent the 49 common targets (Figure 4B). In the diagram, larger nodes indicate higher degree values, signifying a greater likelihood of being a core target. Eighteen targets with elevated Degree Centrality (DC), Closeness Centrality (CC), and Betweenness Centrality (BC) values, surpassing the median, were recognized as critical targets (Table 3). The top 10 targets (STAT3, MMP2, AKT1, JUN, MMP9, PTGS2, CCND1, CASP3, SRC, ICAM1) possessing the highest DC values were chosen as core targets for molecular docking, based on critical targets.

#### 2.5.3. Go Analysis

**Figure 5:**
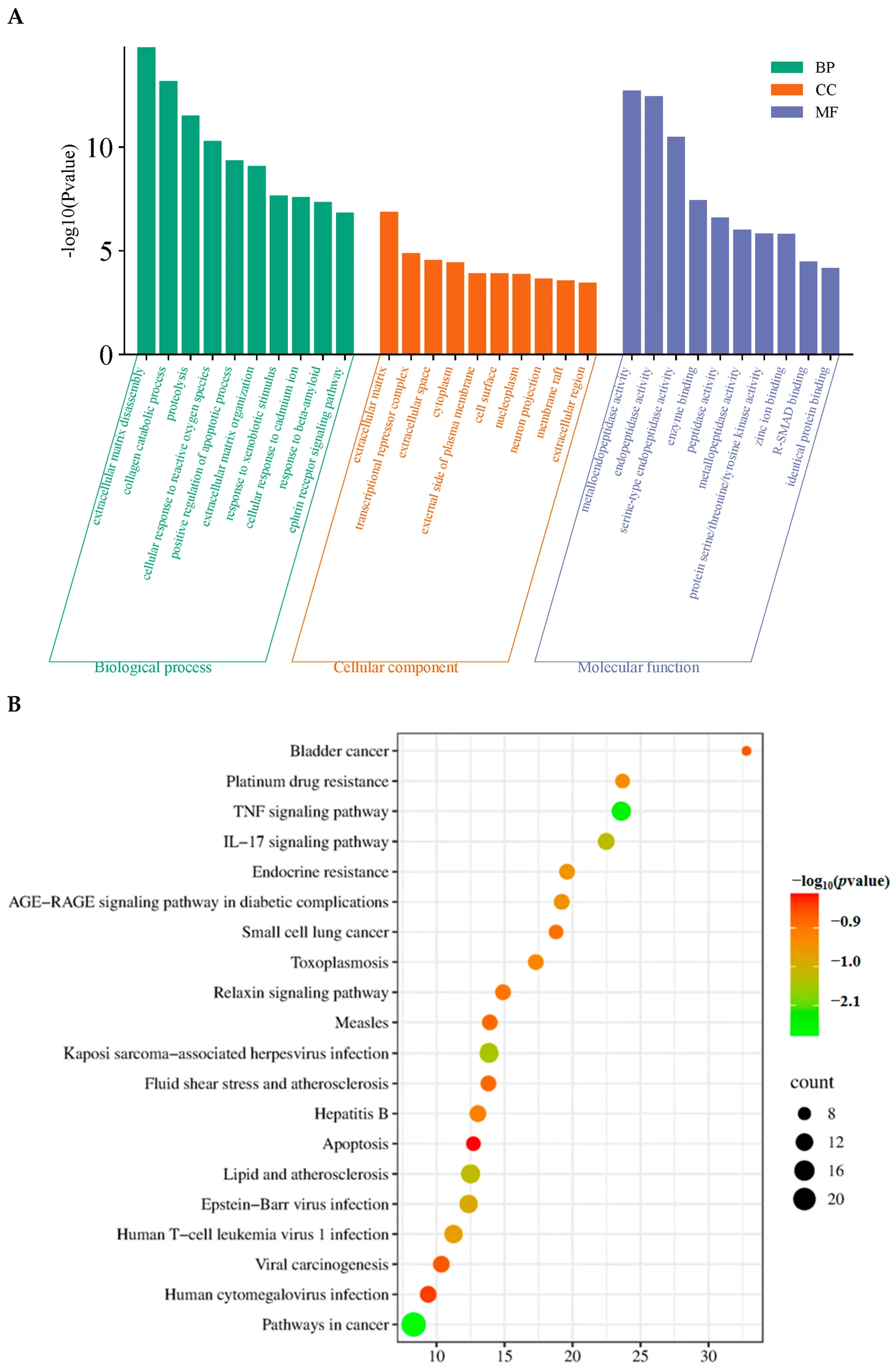
(**A**) Results of GO enrichment analysis and KEGG enrichment analysis. Top 10 biological process (BP) terms, cellular component (CC) terms, and molecular function (MF) terms of GO enrichment analysis are shown as green, orange, and purple bars, respectively. (**B**) The bubble chart of the top 20 pathways based on KEGG enrichment analysis.

A Gene Ontology (GO) analysis was performed on the 49 common targets, revealing a total of 340 annotations distributed among three categories: 237 associated with biological processes (BP), 41 with cellular components (CC), and 62 with molecular functions (MF). The top ten terms in each category, contributing to a sum of 30 terms, are visually represented in a column chart (Figure 5A).

The most highly represented biological process (BP) terms include extracellular matrix disassembly, collagen catabolic processes, proteolysis, cellular response to reactive oxygen species, regulation of the apoptotic process, and extracellular matrix organization. Highly enriched cellular component (CC) terms encompass the extracellular matrix, transcriptional repressor complex [28,29,30], extracellular space, and cytoplasm. Furthermore, molecular function (MF) terms include metalloendopeptidase activity, endopeptidase activity, serine-type endopeptidase activity, enzyme binding, and more.

A KEGG pathway enrichment analysis assessed the 49 common targets, identifying enrichment across 123 pathways. The top 20 pathways were visually analyzed using gene number, fold enrichment, and −log10(*p* value) as evaluation parameters. Figure 5B illustrates the top 20 signal pathways and presents specific information regarding these routes in Table 4. They include pathways such as Pathways in cancer, TNF signaling pathway, Kaposi sarcoma-associated herpesvirus infection, Lipid and atherosclerosis, IL-17 signaling pathway, etc. [31,32,33,34,35,36].

It should be noted that several enriched pathways, including viral infection-associated pathways, m reflect the over-representation of hub genes shared across multiple biological contexts rather than direct evidence of lung-cancer-specific mechanisms. Because the KEGG enrichment analysis was intended solely as an exploratory approach for hypothesis generation, multiple-testing correction was not applied. Accordingly, these KEGG enrichment results serve as exploratory and hypothesis-generating tools to guide future validation, rather than as definitive evidence of direct mechanistic relevance.

### 2.6. Integrated ADMET and Toxicity Profiling of Candidate Peptides

In silico ADMET and toxicity predictions were performed for twelve candidate peptides (FGTQF, RPPGR, WEAWN, FSPDGH, NSQWG, FSTHL, AEWGR, NEYWR, RPFER, STWII, ACGVIGICQ, and IAWTR). Initial screening indicated that five peptides (FGTQF, RPPGR, WEAWN, NSQWG, and ACGVIGICQ) exhibited favorable to acceptable intestinal absorption potential, with low to moderate predicted probabilities (≤0.7) of being classified as HIA+. All peptides showed acceptable distribution and elimination characteristics, including predicted plasma protein binding values ≤90%, suggesting sufficient free drug fractions, consistently low predicted probabilities of blood–brain barrier penetration (≤0.3), and low plasma clearance rates (CLplasma ≤ 5 mL/min/kg), indicative of slow elimination and potential for sustained systemic exposure. With respect to physicochemical and toxicity properties, all peptides except NEYWR exhibited good aqueous solubility (logS ranging from −4 to 0.5), while all peptides except RPPGR showed a low predicted carcinogenicity risk (probability ≤ 0.3). Notably, all twelve peptides demonstrated a low predicted probability of A549 cytotoxicity (≤0.3), indicating favorable non-specific cytotoxic safety profiles. A summary of the predicted ADMET and toxicity results is provided in Table 5, and the criteria used for the in silico evaluation are detailed in Table 6. It is important to note that the A549 cytotoxicity parameter in ADMETlab 3.0 is a safety-oriented prediction that estimates the probability of non-specific cytotoxic liability, rather than a prediction of selective anticancer activity. An “Excellent” rating (probability ≤ 0.3) therefore indicates a low risk of non-specific cytotoxicity, representing a favorable safety profile for candidate peptides. The anticancer potential of these peptides was assessed through dedicated anticancer peptide prediction tools (PeptideRanker, AntiCP, iACP, and ACPred) in the preceding screening steps, and the ADMET evaluation served as a complementary safety filter rather than a predictor of anticancer efficacy. Based on an integrated evaluation of the predicted ADME and toxicity profiles, four peptides (FGTQF, WEAWN, NSQWG, and ACGVIGICQ) were ultimately selected as lead candidates for subsequent molecular docking analysis and in vitro experimental validation.

To provide complementary pharmacokinetic and safety information more specifically tailored to peptide molecules, all twelve candidate peptides were further evaluated using pepADMET, a peptide-specific ADMET prediction platform trained exclusively on peptide datasets. Unlike ADMETlab 3.0, which was developed primarily for drug-like small molecules, pepADMET is specifically designed for peptide ligands and therefore provides complementary predictions within a more appropriate applicability domain for the peptide candidates investigated in this study. It should be noted that the selection of FGTQF, WEAWN, NSQWG, and ACGVIGICQ for subsequent molecular docking and experimental validation was based on the integrated ADMETlab 3.0 evaluation described above, and the pepADMET analysis was conducted as a complementary peptide-specific assessment to provide additional pharmacokinetic and safety characterization of all twelve candidates. The results are summarized in Table 7.

All twelve peptides were predicted to exhibit low oral bioavailability (F−, <20%), consistent with the generally poor gastrointestinal absorption of short natural peptides and in agreement with the intestinal absorption profiles obtained from ADMETlab 3.0. All twelve peptides were predicted as BBB+ by pepADMET; however, given the well-recognized challenges associated with peptide penetration across the blood–brain barrier in vivo, this prediction should be interpreted with caution and requires further experimental validation. With respect to plasma stability, NSQWG (251.02 min), ACGVIGICQ (254.38 min), and WEAWN (159.33 min) exhibited relatively longer predicted half-lives in human blood than the remaining peptides, providing additional support for their prioritization as candidate peptides. Although WEAWN showed a relatively higher predicted toxicity probability (0.81) than the other prioritized peptides, its predicted cytotoxicity liability remained negligible (0.01), suggesting that any predicted toxicity may not primarily arise from direct cytotoxic mechanisms. NSQWG and ACGVIGICQ exhibited low predicted toxicity probabilities (0.34 and 0.23, respectively) together with negligible predicted cytotoxicity liabilities (0.00), suggesting favorable safety profiles in silico. All peptides exhibited negative LogD7.4 values (−0.32 to −2.02), consistent with their relatively hydrophilic physicochemical properties and in agreement with the aqueous solubility predictions generated by ADMETlab 3.0.

Collectively, the pepADMET analysis provides peptide-specific pharmacokinetic and safety information that complements the original ADMETlab 3.0 evaluation and supports the overall candidate prioritization strategy adopted in this exploratory study.

### 2.7. Molecular Docking Study

It was determined through protein–protein interaction (PPI) analysis that STAT3, MMP2, AKT1, JUN, MMP9, PTGS2, CCND1, CASP3, SRC, and ICAM1 occupied the top 10 positions based on their degree values. These ten targets are well-recognized cancer-associated proteins involved in diverse biological processes related to tumor development, including cell proliferation, apoptosis, and extracellular matrix remodeling. They were computationally prioritized as candidate docking targets based on their topological prominence in the PPI network and selected for exploratory in silico molecular docking analysis to generate hypotheses regarding potential peptide–protein interactions rather than to establish experimentally validated peptide–target interactions or mechanisms of action. The docking parameters for each target protein, including PDB accession codes, grid box center coordinates, grid dimensions, and grid spacing, are summarized in Table 8. All docking calculations were performed using a uniform grid dimension of 126 × 126 × 126 points, with individual grid spacing values determined based on the structural characteristics of each target protein.

**Figure 6:**
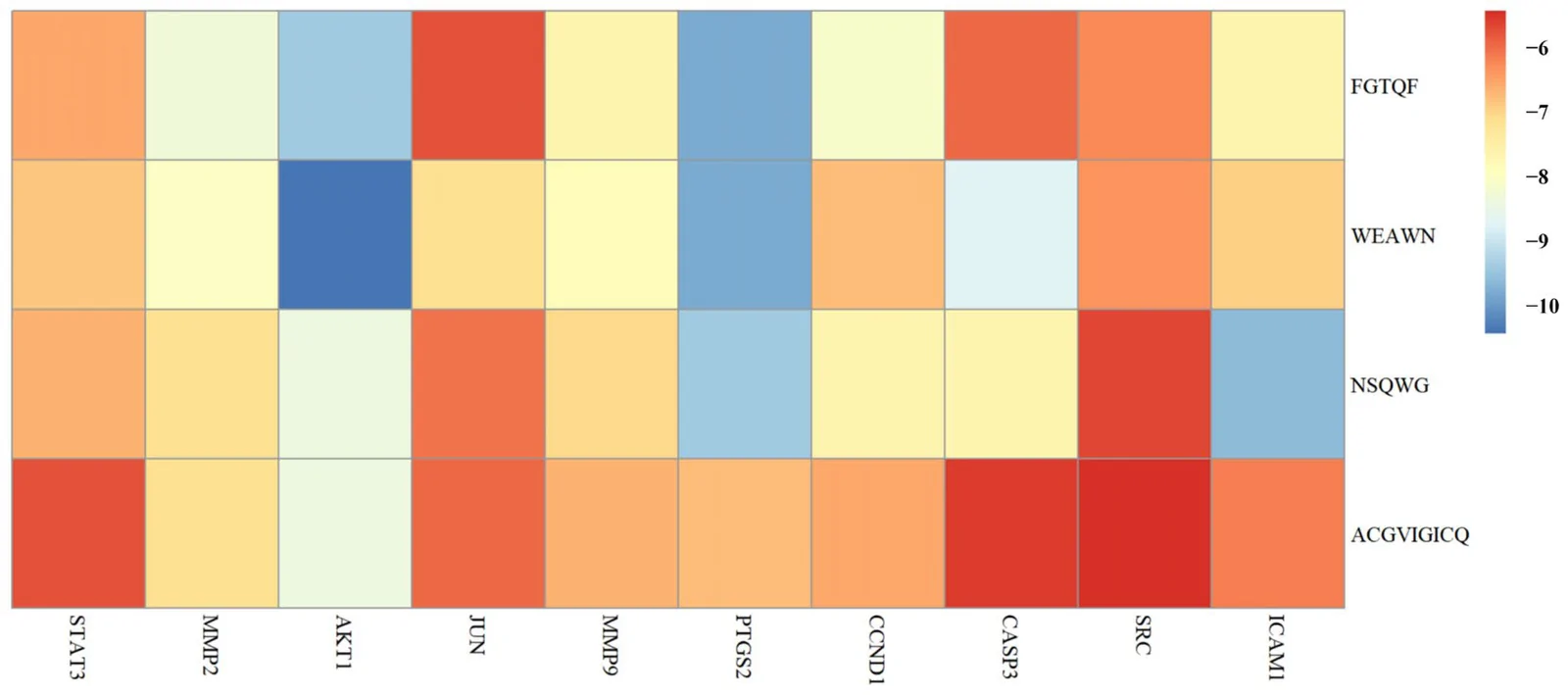
Heat map of molecular docking score. Binding energy (kcal/mol) of key targets and active peptide of toad poison.

**Figure 7:**
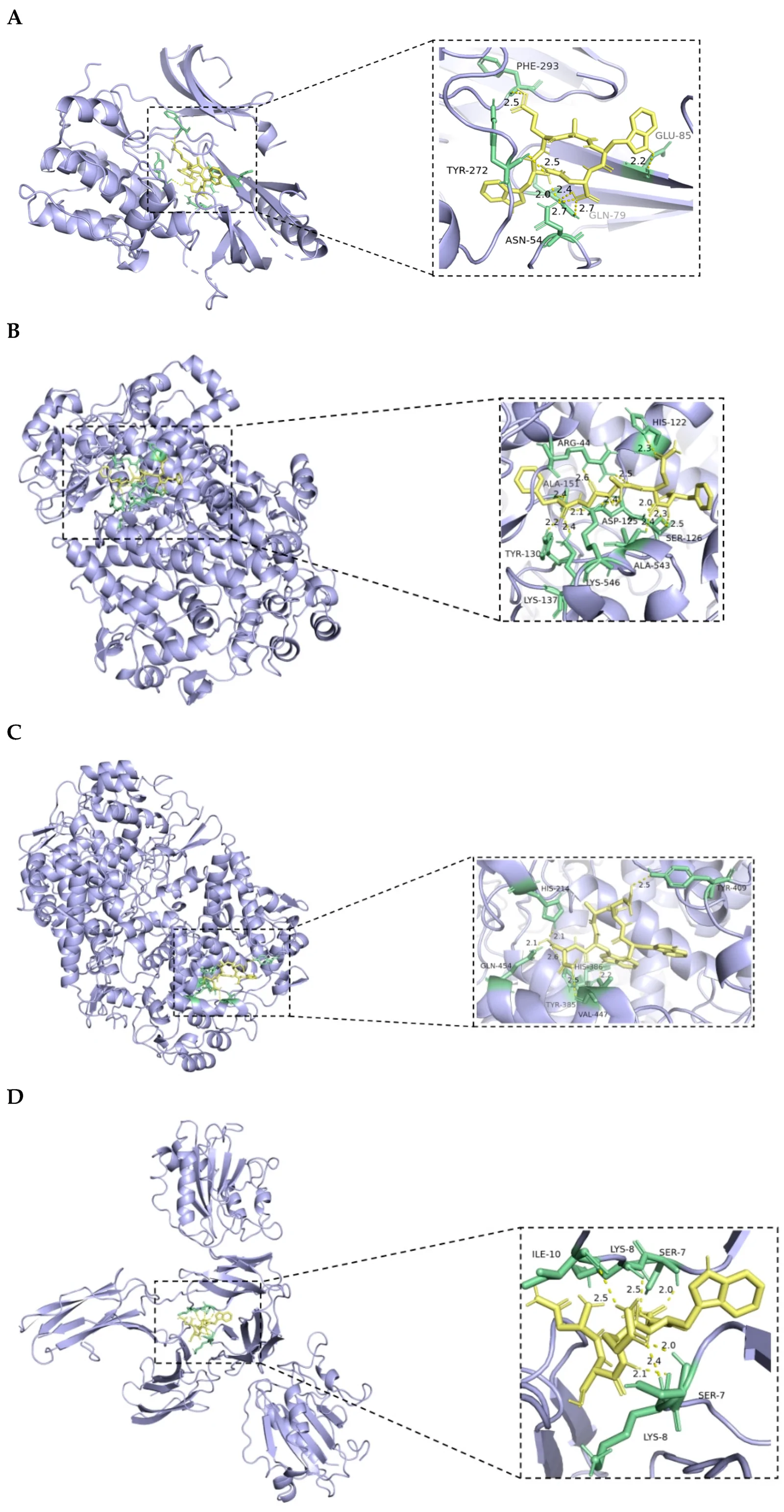

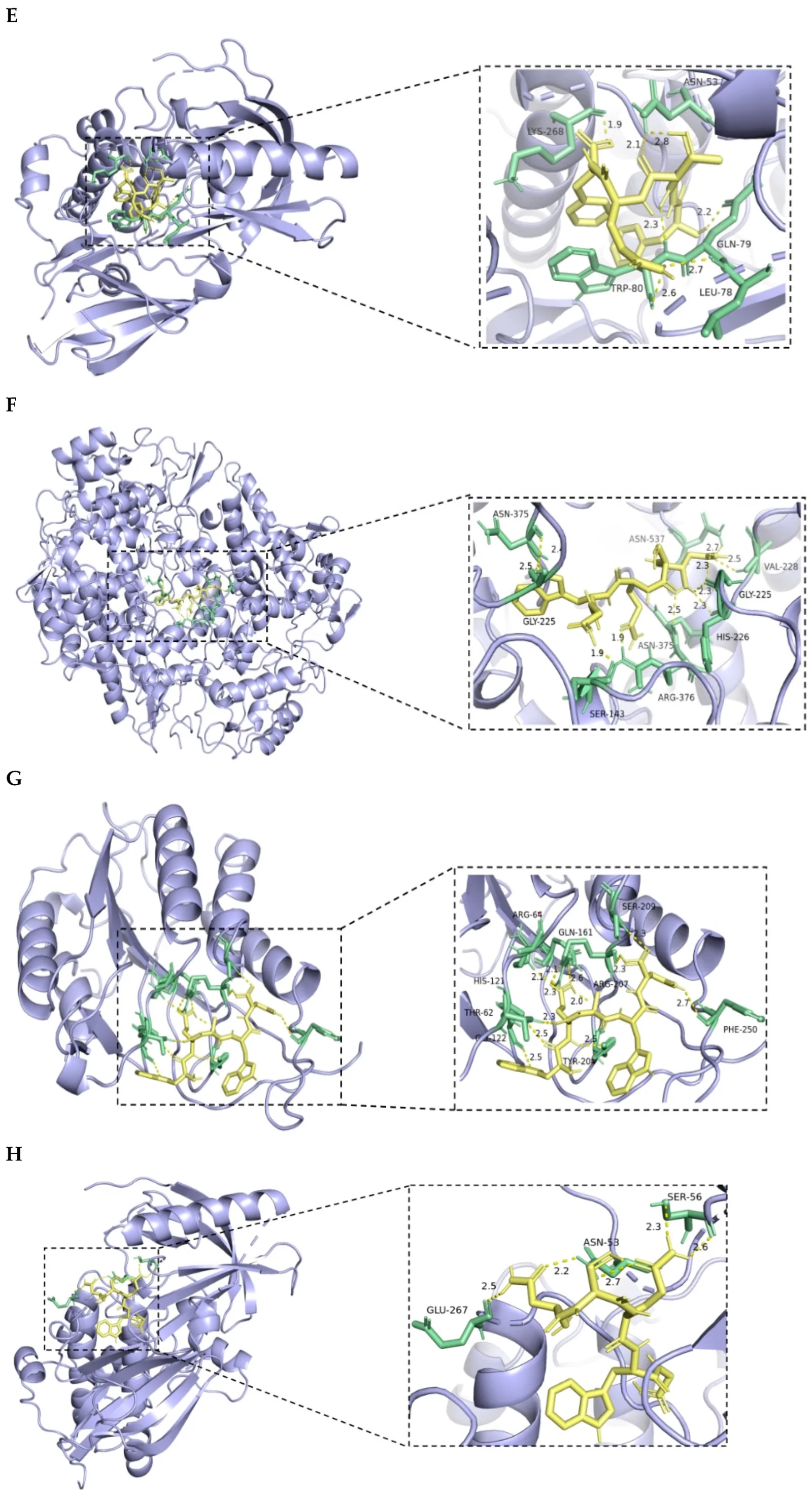

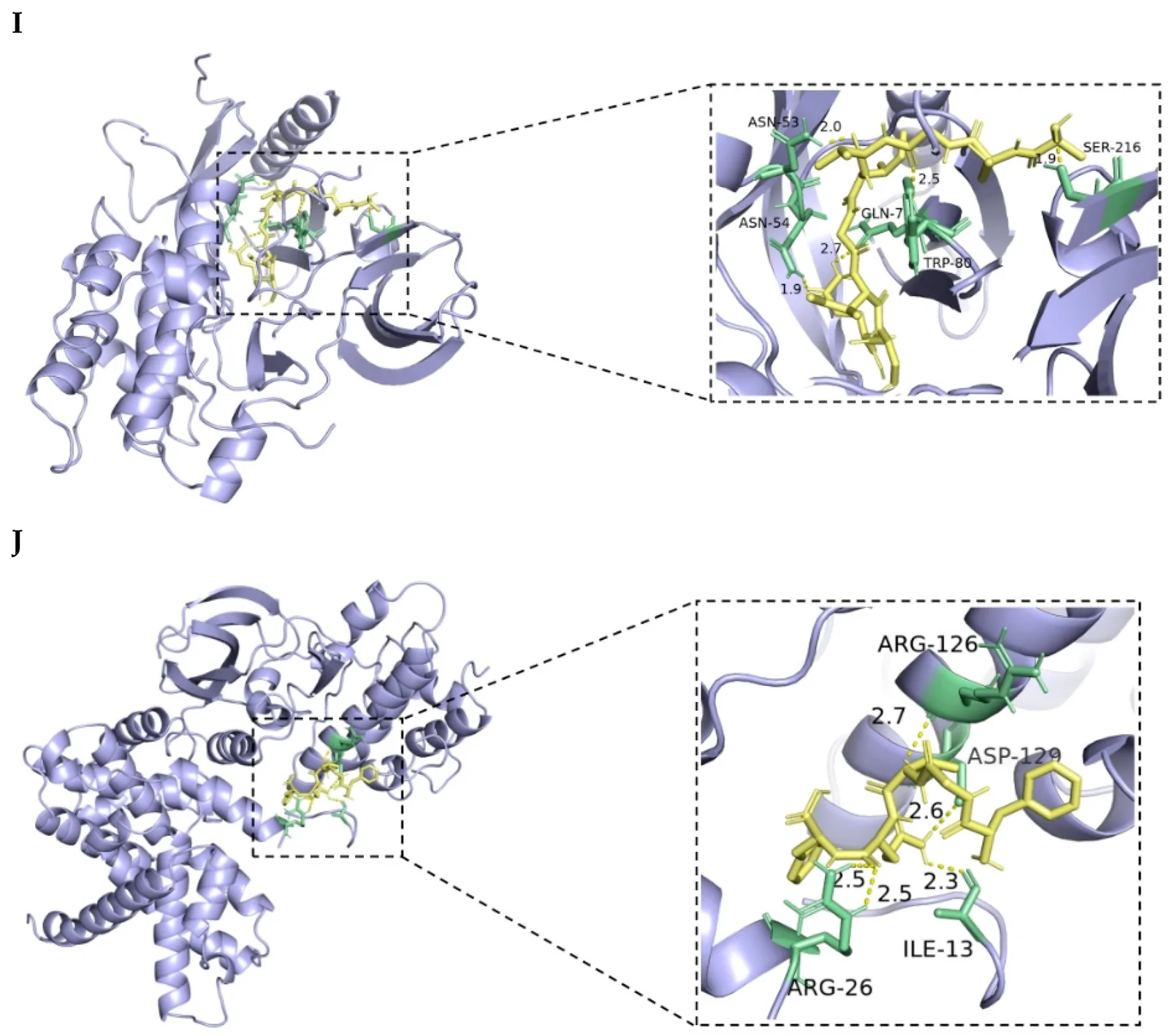
Docking patterns of key targets and anticancer peptide. (**A**) WEAWN-AKT1; (**B**) FGTQF-PTGS2; (**C**) WEAWN-PTGS2; (**D**) NSQWG-ICAM1; (**E**) FGTQF-AKT1; (**F**) NSQWG-PTGS2; (**G**) WEAWN-CASP3; (**H**) NSQWG-AKT1; (**I**) ACGVIGICQ-AKT1; (**J**) FGTQF-CCND1. Yellow denotes the peptide ligand, green highlights the key interacting amino acid residues, and blue-purple represents the overall protein structure.

The molecular docking results revealed that the four active peptides exhibited binding energies below −5 kcal/mol when interacting with core proteins, indicating spontaneous ligand-receptor binding. A heat map was generated based on these results (Figure 6). These outcomes provide preliminary computational evidence of potential interactions between the candidate peptides and cancer-associated proteins, though experimental validation of target engagement remains necessary. The optimal docking of the ten lowest-binding-energy receptor-ligand pairs was visualized (Figure 7).

The docking analysis identified potential π–π stacking interactions and hydrogen-bonding patterns between the candidate peptides and their putative target proteins [37,38,39]. These interactions are presented as preliminary computational observations intended for hypothesis generation rather than as evidence of experimentally validated binding. The observed structural interaction patterns warrant further experimental verification in future studies. It should be acknowledged that AutoDock Vina’s scoring function was originally developed for small-molecule ligands and may have limited accuracy for the flexible 5–9-residue peptides investigated in this study. Additionally, JUN and CCND1, two of the ten selected docking targets, lack well-characterized canonical druggable pockets, which may reduce the reliability of the docking predictions for these specific targets. The docking results for all targets should therefore be interpreted as preliminary computational estimates rather than as quantitatively validated binding predictions.

### 2.8. Toad Poison Active Peptide Decreases Proliferation of A549 Cells

**Figure 8:**
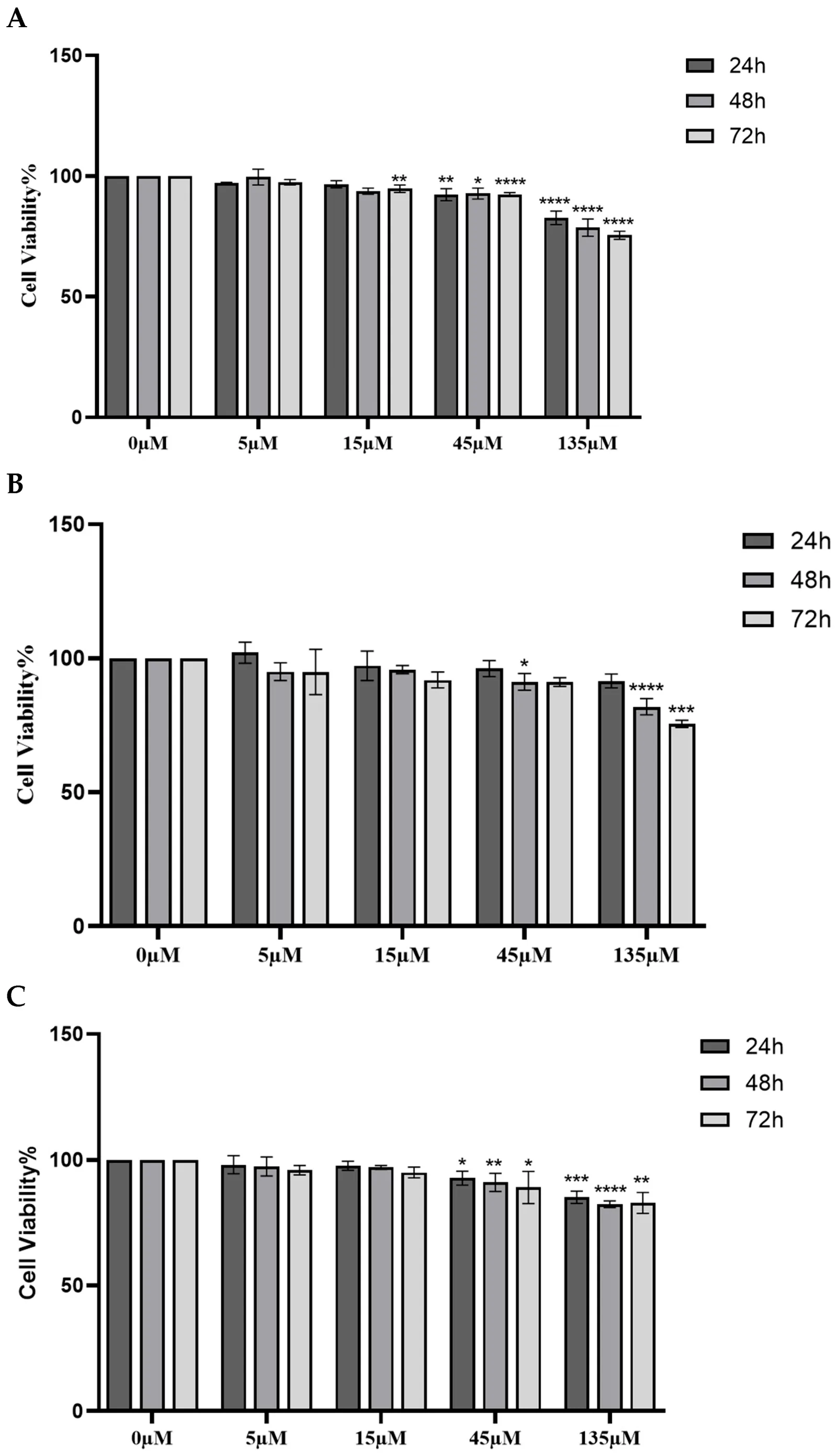
Inhibition of A549 cell viability treated by (**A**) WEAWN, (**B**) NSQWG, (**C**) ACGVIGICQ, trials (* *p* < 0.05, ** *p* < 0.01, *** *p* < 0.001, **** *p* < 0.0001).

The effects of the active peptides on the viability and proliferative activity of lung cancer cells were evaluated using the MTT assay after 24, 48, and 72 h of treatment. The results demonstrated that three of the four predicted active peptides derived from toad poison (WEAWN, NSQWG, and ACGVIGICQ) produced a modest but statistically significant, dose-dependent inhibition of cell viability and proliferation compared with the untreated control (Figure 8).

## 3. Discussion

In the present study, an integrated and efficient antitumor peptide screening strategy was established to systematically explore bioactive peptides from toad poison, a complex animal-derived traditional medicine. By combining proteomics-based protein identification with in silico enzymatic digestion, a large virtual peptide library comprising 2117 peptide sequences was generated from 135 identified proteins. This strategy substantially expanded the accessible peptide space while effectively circumventing the technical challenges associated with direct peptide isolation and purification from complex natural matrices.

The stepwise virtual screening workflow enabled rational prioritization of candidate peptides with high biological relevance. Initial screening using PeptideRanker facilitated rapid assessment of general bioactivity potential, whereas subsequent cross-validation using multiple anticancer peptide prediction platforms (AntiCP, ACPred, and iACP) reduced the inherent bias associated with individual algorithms. Furthermore, incorporation of ADMET-based evaluation refined candidate selection by excluding peptides with unfavorable predicted toxicity or pharmacokinetic properties, thereby improving the translational relevance of the screened peptides. It should be clarified that the A549 cytotoxicity metric in ADMETlab 3.0 was employed as a safety filter reflecting non-specific cytotoxic liability, rather than a predictor of anticancer efficacy, and the observed in vitro antiproliferative activity and the ‘Excellent’ A549 cytotoxicity rating therefore reflect two distinct and complementary dimensions: selective antiproliferative potential and non-specific cytotoxic safety, respectively. It should also be acknowledged that SwissTargetPrediction, upon which the network pharmacology analysis in this study is based, was developed primarily for drug-like small molecules and has not been specifically optimized for peptide ligands. Accordingly, the network pharmacology, PPI, and enrichment analyses presented here should be regarded as exploratory and hypothesis-generating, providing a preliminary computational basis for future experimental target validation rather than definitive mechanistic evidence. The development of peptide-specific platforms capable of proteome-wide target prediction remains an important challenge in this field, and future studies should incorporate such approaches as they become available. Collectively, this multi-layered strategy represents a robust, reproducible, and efficient framework for antitumor peptide discovery from complex natural sources.

Molecular docking analysis provided complementary insights into the potential biological relevance of the identified peptides by suggesting favorable interactions with cancer-related target proteins. The computational screening identified several cancer-associated proteins as candidate binding partners, providing a preliminary basis for future mechanistic investigations. While docking results cannot substitute for direct mechanistic validation, these computational findings, together with the observed in vitro antiproliferative activity, highlight the potential value of integrating in silico approaches with experimental validation during early-stage peptide screening. These computational findings provide a preliminary rationale for future mechanistic investigations, including flow cytometry-based apoptosis detection (Annexin V/PI staining), caspase activity analysis, reactive oxygen species (ROS) measurement, and mitochondrial membrane potential assessment, to experimentally investigate the potential mechanisms underlying the observed antiproliferative effects.

Based on the computational screening results, four peptides (FGTQF, WEAWN, NSQWG, and ACGVIGICQ) were selected for in vitro experimental validation. Among these, three peptides (WEAWN, NSQWG, and ACGVIGICQ) demonstrated modest but statistically significant antiproliferative activity against A549 lung cancer cells in a dose- and time-dependent manner, providing initial experimental support for the virtual screening strategy. It should be noted that none of the tested peptides reduced cell viability below 50% at the highest concentration evaluated (135 μM); therefore, these peptides should be regarded as preliminary lead candidates with initial antiproliferative activity rather than as fully validated or potent anticancer agents. The lack of antiproliferative activity observed for FGTQF may be partially explained by its relatively short predicted plasma half-life in human blood (HBN T1/2: 22.3 min) as assessed by the peptide-specific pepADMET platform, suggesting potential instability under experimental conditions. In contrast, WEAWN, NSQWG, and ACGVIGICQ, which demonstrated initial antiproliferative activity, exhibited comparatively more favorable predicted plasma stability (HBN T1/2: 159.3, 251.0, and 254.4 min, respectively). These observations retrospectively suggest that plasma stability may be an important determinant of antiproliferative activity among the candidate peptides evaluated in this study, and that peptide-specific ADMET tools may provide more relevant pharmacokinetic predictions for future candidate prioritization. Furthermore, the discrepancy between FGTQF’s favorable docking scores and its lack of in vitro activity underscores the inherent limitations of AutoDock Vina’s scoring function when applied to flexible peptide ligands, and reinforces the importance of experimental validation as an essential complement to computational screening. Multiple factors beyond binding affinity, including peptide stability, cellular uptake, and off-target interactions, may collectively influence the observed in vitro activity, and the docking model employed in this study should be regarded as a hypothesis-generating tool rather than a validated predictive model.

Compared with conventional bioactivity-guided fractionation strategies, which are typically labor-intensive, time-consuming, and dependent on extensive purification procedures, the screening strategy employed in this study markedly improves efficiency while reducing experimental cost and resource consumption. More importantly, this workflow enables systematic exploration of the previously unexplored peptidome within toad poison, thereby extending current research beyond its well-studied small-molecule constituents. These findings highlight the potential of animal-derived traditional medicines as underexplored reservoirs of bioactive peptides with novel pharmacological activities.

It is worth emphasizing that the present study was designed as an exploratory, methodology-driven investigation aimed at establishing an efficient discovery framework for bioactive peptides from complex natural sources rather than identifying highly potent anticancer agents. Accordingly, the in vitro cytotoxicity assay was included as an initial experimental verification of the computational predictions. Comprehensive pharmacological characterization, including IC_50_ determination and extended dose–response analysis, was beyond the scope of the current investigation. Further studies involving structural optimization, selectivity assessment against normal cells, and evaluation of in vivo efficacy and pharmacokinetic properties will be necessary to fully assess the therapeutic potential of these preliminary lead peptides.

Furthermore, it should be acknowledged that the peptide candidates identified in this study were generated through in silico digestion and have not yet been experimentally verified. Future peptidomic analyses of gastrointestinal digests of toad poison proteins are warranted to determine whether these predicted peptides can be generated under physiologically relevant conditions. Furthermore, the STRING protein–protein interaction network was constructed using a medium confidence threshold (score ≥ 0.4), which may have introduced lower-confidence interactions and potentially influenced network edge counts and centrality metrics. Future studies should consider applying higher confidence thresholds or cross-validating key interactions using experimentally curated databases to improve the reliability of the network topology analysis. It should also be noted that peptide conformers used for molecular docking were generated using a single energy minimization step without comprehensive conformational sampling, which represents a methodological limitation of the current docking workflow. Future studies should incorporate more rigorous conformational sampling strategies to improve the reliability of peptide docking predictions.

Another limitation of the present study is the exclusive use of the A549 cell line for in vitro validation. While A549 is a well-established model of non-small cell lung cancer and is consistent with our network pharmacology focus on lung cancer-related targets, the use of a single cancer cell line does not allow conclusions regarding cancer selectivity or potential toxicity toward normal cells. Future studies should include additional cancer cell lines and non-tumorigenic cell models to comprehensively evaluate the selectivity, safety profile, and therapeutic potential of the identified peptides.

It should also be noted that no positive control was included in the MTT assay, which represents a methodological limitation of the current study. The primary objective of the cell-based experiments was to determine whether the computationally prioritized peptides exhibited measurable antiproliferative activity relative to untreated controls. Although the inclusion of a positive control such as cisplatin or doxorubicin would have strengthened the experimental design and facilitated potency comparison with established anticancer agents, the observed antiproliferative effects of the identified peptides remained reproducible and statistically significant under the conditions tested. Future pharmacological evaluations should incorporate appropriate positive controls to enable more rigorous assessment of peptide activity. It should also be acknowledged that the candidate targets identified through network pharmacology analysis, including STAT3, AKT1, and JUN, are primarily intracellular proteins. Whether the identified peptides are capable of cellular internalization or instead exert their biological effects through extracellular mechanisms remains to be experimentally determined. Future target engagement studies, such as Western blot analysis, surface plasmon resonance, or caspase activity assays, are warranted to substantiate the preliminary computational observations reported in this study.

## 4. Conclusions

This study provides a systematic exploration of protein-derived peptide candidates from toad poison using an integrated proteomics- and bioinformatics-guided discovery framework. Three peptides (WEAWN, NSQWG, and ACGVIGICQ) were identified as preliminary lead candidates possessing initial antiproliferative activity against A549 lung cancer cells. Although the absence of a positive control limits direct comparison with established anticancer compounds, these results provide preliminary experimental support for the computational predictions. Furthermore, while further targeted analytical investigations, such as LC-MS/MS peptidomic analysis, are necessary to confirm the presence of these specific sequences in native poison, and additional studies—including mechanistic evaluation, selectivity assessment, and in vivo validation—are warranted to fully substantiate these initial observations, the present findings provide preliminary insights into the antiproliferative potential of toad poison protein-derived peptide candidates warranting further pharmacological investigation. Finally, this work demonstrates the value of integrated computational–experimental strategies for the exploratory discovery of potential bioactive peptides from complex animal-derived traditional medicines.

## 5. Materials and Methods

### 5.1. Materials and Reagents

Trypsin (proteomics grade), ammonium bicarbonate, Triton X-100, Iodoacetamide (IAA), and Thiazolyl Blue Tetrazolium Bromide (MTT) were procured from Sigma-Aldrich (St. Louis, MO, USA). Formic acid (Optima LCMS) was sourced from Thermo Fisher Scientific (Waltham, MA, USA) and acetonitrile (gradient grade) from Merck KGa (Darmstadt, Germany). Millipore’s Milli-Q Gradient A10 system provided water (Schwalbach, Germany). Dithiothreitol (DTT), Urea, and Thiourea were obtained from Hushi (Shanghai, China). RPMI 1640 was acquired from Procell (Wuhan, China), Fetal bovine serum from TianHang (Huzhou, China), and PBS and Antibiotics (Penicillin-Streptomycin Liquid (100×)) from Solarbio (Beijing, China). Modified Bradford Protein Assay Kit was procured from Sangon Biotech (Shanghai, China). In this study, all four peptide markers were synthesized by China Peptides Co., Ltd. (Shanghai, China). For ACGVIGICQ, which contains two cysteine residues, the peptide was synthesized in the reduced form with free thiol groups.

In this study, the sample information is summarized in Table 9.

### 5.2. Sample Preparation

#### 5.2.1. Toad Poison Protein Extraction

Accurately weigh approximately 250 mg of the toad poison sample powder, combine it with the protein lysis solution (Urea 21.0 g, Thiourea 7.6 g, TritonX-100 50 μL, and add ammonium bicarbonate solution at a concentration of 50 mM to reach a volume of 50 mL). Perform ultrasonic extraction for 30 min, followed by overnight incubation. Subsequently, centrifuge the mixture at 4 °C for 15 min at 12,000 rpm. Collect the supernatant to obtain the protein sample solution.

#### 5.2.2. Determination of Protein Concentration

The Bradford method and a dedicated protein concentration determination kit assessed the protein concentration.

Standard Solution Preparation: Aspirate 1.00 mg/mL of BSA standard protein in volumes of 0, 50, 100, 150, 200, 250, and 300 μL into a 2 mL centrifuge tube. Subsequently, dilute each aliquot with purified water (1000, 950, 900, 850, 800, 750, and 700 μL, respectively) to achieve concentrations of 0, 0.05, 0.10, 0.15, 0.20, 0.25, and 0.30 mg/mL BSA standard protein solution. Various concentrations of BSA standard protein and a 100-fold diluted toad poison protein solution (20 μL each) were pipetted into distinct wells of a 96-well plate. Subsequently, 200 μL of Bradford’s working solution was added to each well, and the absorbance at 595 nm was measured using a microplate reader.

#### 5.2.3. Reductive Alkylation

Begin by taking 250 μg of the protein sample and adding 1M DTT to achieve a final DTT concentration of 10 mM. Allow the reaction to proceed at room temperature for 3 h. Subsequently, introduce an appropriate quantity of 1 M IAA to attain a final IAA concentration of 50 mM. Let the reaction continue at room temperature in darkness for 1 h. Following this step, introduce a 50 mM ammonium bicarbonate solution to reduce the urea concentration to below 1 M.

#### 5.2.4. Trypsin Digestion

Introduce trypsin at a protein-to-trypsin ratio of 100:1 for trypsin digestion. Conduct overnight digestion at 37 °C within a consistently maintained temperature incubator. After this, reintroduce trypsin at a protein-to-trypsin ratio of 100:1, extending the digestion at 37 °C in a continuous temperature incubator for 1 h. Add a suitable formic acid (FA) to achieve a final FA concentration of 0.5%, concluding the digestion reaction. After digestion, utilize a Sep-Pak C18 column for the desalting process.

### 5.3. Nano LC-MS/MS Analysis

The processed samples underwent analysis using a nano-flow liquid chromatograph (EASY-nLC 1000, Thermo Scientific, San Jose, CA, USA) coupled with high-resolution mass spectrometry (Orbitrap-Fusion, Thermo Scientific, San Jose, CA, USA) under the specified conditions:

EASY-nLC conditions: Desalination and enrichment on a Thermo Acclaim PepMap C18 column (75 μm × 2 cm, 3 μm, Thermo Scientific, San Jose, CA, USA), followed by separation on a Thermo Acclaim PepMap C18 column (75 μm × 15 cm, 3 μm, Thermo Scientific, San Jose, CA, USA) at a flow rate of 300 nl/min. Mobile phase A consisted of an aqueous 0.1% formic acid solution, while mobile phase B comprised an aqueous 0.1% formic acid solution containing 99.9% acetonitrile. The elution gradient was as follows: 0–1 min, 1% B→6% B; 1–96 min, 6% B→22% B; 96–113 min, 22% B→30% B; 113–117 min, 30% B→95% B; 117–120 min, 95% B. The injection volume was 1 μL.

In this study, high-resolution mass spectrometry was conducted under the following optimized conditions: The Nanospray Flex ion source was employed, operating in positive ion mode with a spray voltage set to 1800 V; the ion transport capillary temperature was maintained at 275 °C, and the S-Lens transport efficiency was fine-tuned to 60%. The primary mass spectrometry utilized an Orbitrap mass analyzer with a resolution of 60,000 and an acquisition range spanning 350–1550 (*m*/*z*). For secondary mass spectrometry, an Orbitrap mass analyzer in Rapid Scan mode was employed for scanning, Top speed data-dependent mode for precursor ion selection, HCD mode for fragmentation, and a fragmentation energy NCE set to 40%.

### 5.4. Mass Spectrometry Data Analysis

The data underwent processing and analysis utilizing PEAKS Studio software (version 8.5, Bioinformatics Solutions Inc., Waterloo, ON, Canada). The procedures included data processing and spectrum analysis, encompassing de novo sequencing and database search. The specified parameters were Trypsin enzyme digestion, with no fixed modification, and variable modifications set to HYP, Deamidation, Oxidation, and Acetylation. A parent mass error tolerance of 15.0 ppm, fragment mass error tolerance of 0.02 Da, and a maximum of 3 allowed missed cleavages were applied. De novo score threshold was set at 15, and the filter charge ranged from 2 to 8. Establishing a false discovery rate (FDR) of <1% ensured the identification of results with confidence. Protein sequences were chosen based on their protein content and plausibility for subsequent analysis.

### 5.5. Results of Online Enzyme Cutting

The protein sequences obtained were subjected to in silico enzymatic digestion using the ExPASy PeptideCutter website (https://web.expasy.org/peptide_cutter/, accessed on 15 October 2025). Both pepsin and trypsin cleavage rules were applied to each protein sequence to maximize peptide sequence coverage. The peptide fragments generated by the two enzymes were combined, duplicate sequences were removed, and peptides ranging from 5 to 40 amino acids in length were retained for subsequent analysis.

It should be noted that the in silico digestion strategy was employed as a peptide discovery approach to explore potentially bioactive fragments encrypted within poison proteins, rather than to characterize the endogenous peptide composition of native toad poison.

### 5.6. Prediction of the Anticancer Activity of Toad Poison Peptides

PeptideRanker (http://distilldeep.ucd.ie/PeptideRanker/, accessed on 20 October 2025) was used to evaluate the probability of biological activity for each peptide candidate. According to the recommendations of the tool developers [40], peptides with scores greater than 0.5 are considered more likely to exhibit biological activity. Therefore, a threshold of 0.5 was employed as the initial screening criterion in the present study.

Following Online Enzyme Cutting, the identified peptide sequences underwent bioactivity scoring using PeptideRanker. The peptide sequences were formatted in FASTA and utilized as input for predicting anticancer properties through three machine-learning-based prediction servers: AntiCP (https://webs.iiitd.edu.in/raghava/anticp/index.html, accessed on 28 October 2025), ACPred (http://codes.bio/acpred/, accessed on 6 November 2025), and Iacp (https://lin.uestc.edu.cn/server/iACP, accessed on 8 November 2025). Venny 2.1.0 (https://bioinfogp.cnb.csic.es/tools/venny/, accessed on 11 November 2025) was employed to generate a Venn diagram, illustrating the unique, dual, and multifunctional peptide candidates from each prediction online bioinformatics program. To improve prediction reliability and reduce false-positive predictions, only peptide sequences consistently classified as anticancer (active) by all three prediction servers were retained as final candidates. This consensus-based filtering strategy was adopted to increase confidence in the selected peptides and mitigate biases associated with individual prediction algorithms. Subsequently, Toxinpred (https://webs.iiitd.edu.in/raghava/toxinpred/index.html, accessed on 15 November 2025) assessed the cytotoxicity of the peptides. Additionally, BIOPEP (https://biochemia.uwm.edu.pl/, accessed on 16 November 2025) was utilized to verify whether the screened peptides had been previously reported.

### 5.7. Results of Network Pharmacology Analysis

#### 5.7.1. Screening the Targets of Active Peptide and Lung Cancer

Furthermore, SwissTargetPrediction (http://www.swisstargetprediction.ch/, accessed on 21 November 2025) was utilized to predict putative molecular targets for the candidate peptides. It is acknowledged that SwissTargetPrediction was designed primarily for small-molecule drug candidates within Lipinski chemical space, and its predictive applicability to short peptides may be limited due to differences in molecular size, hydrogen bond capacity, and charge distribution. Accordingly, the predicted targets should be interpreted as exploratory and hypothesis-generating rather than as definitive mechanistic evidence. Lung cancer-associated targets were collected from five databases: OMIM (https://omim.org/, accessed on 24 November 2025), DrugBank (https://go.drugbank.com/, accessed on 24 November 2025), GeneCards (https://www.genecards.org/, accessed on 24 November 2025), the Therapeutic Target Database (TTD, https://db.idrblab.net/ttd/, accessed on 24 November 2025), and DisGeNET (https://www.disgenet.org/, accessed on 24 November 2025) using the following keywords: “lung cancer”, “Small Cell Lung Cancer”, “Non-Small Cell Lung Cancer”, and “Malignant neoplasm of the lung”. Duplicate entries were subsequently removed to establish the lung cancer target dataset.

#### 5.7.2. Construction of PPI Network

A Venn diagram was employed to identify gene targets that intersected between the two datasets. The common targets within this intersection were then input into the STRING database (https://cn.string-db.org/, accessed on 26 November 2025) to retrieve information on the protein interaction network. Organism screening conditions were set to “Homo sapiens,” with a minimum required interaction score of “medium confidence ≥ 0.4.” The obtained protein–protein interaction (PPI) information was visualized using Cytoscape 3.8.2, forming a comprehensive PPI network. Essential target proteins were selected based on the criterion that their degree centrality (DC), closeness centrality (CC), and betweenness centrality (BC) exceeded the median. Subsequently, the critical target proteins from the network pharmacology screening were ranked according to the degree value, with the top 10 identified as core targets.

#### 5.7.3. GO Enrichment and KEGG Pathway Analysis

To investigate the biological functions of potential targets in lung cancer, we conducted analyses on intersecting genes using Gene Ontology (GO) and Kyoto Encyclopedia of Genes and Genomes (KEGG), employing the DAVID database (https://david.ncifcrf.gov/, accessed on 26 November 2025). The GO analysis allowed for the scrutiny of biological processes (BP), cellular components (CC), and molecular functions (MF). Simultaneously, KEGG enrichment analysis aimed to identify pivotal signalling pathways implicated in biological processes. The resultant GO and KEGG data were then uploaded to the Bioinformatics platform (https://www.bioinformatics.com.cn/, accessed on 26 November 2025) for comprehensive visual analysis.

### 5.8. ADMET Properties Prediction

To further evaluate the pharmacokinetic characteristics and safety profiles of the candidate anticancer peptides, in silico ADME and toxicity predictions were performed using the ADMETlab 3.0 platform [41] (https://admetlab3.scbdd.com/, accessed on 2 January 2026). Human intestinal absorption (HIA) was predicted to evaluate absorption potential, while plasma protein binding (PPB) and blood–brain barrier (BBB) penetration were used to characterize distribution properties. Plasma clearance (CLplasma) was assessed to reflect metabolic and excretory behavior. In addition, key physicochemical parameters, including the logarithm of aqueous solubility (logS) and the logarithm of the n-octanol/water partition coefficient (logP), were predicted to describe solubility and lipophilicity. Toxicological profiles were further assessed by predicting carcinogenicity and A549 cytotoxicity [42,43,44]. These predicted ADME and toxicity parameters were integrated as supporting evidence to characterize the developability of the screened peptides, providing a rational basis for subsequent molecular docking analysis and in vitro experimental validation.

To complement the ADMETlab 3.0 predictions and provide pharmacokinetic and safety information using a prediction model specifically developed for peptide molecules, all twelve candidate peptides were additionally evaluated using pepADMET (https://pepadmet.ddai.tech/, accessed on 28 June 2026), a peptide-specific ADMET prediction platform trained exclusively on peptide datasets. The following prediction endpoints were assessed: oral bioavailability (F, classified as F+ if ≥20% or F− if <20%), blood–brain barrier penetration (BBB, classified as BBB+ or BBB−), predicted plasma half-life in human blood under natural peptide conditions (HBN T1/2, reported in minutes), overall toxicity classification (probability of toxic or non-toxic), predicted cytotoxicity liability, and the distribution coefficient at physiological pH (LogD7.4). Particular attention was given to predicted plasma stability (HBN T1/2), overall toxicity probability, cytotoxicity liability, and physicochemical properties (LogD7.4), whereas the predicted low oral bioavailability (F−) was interpreted as a common pharmacokinetic characteristic of short natural peptides rather than a criterion for candidate prioritization. It should be noted that the pepADMET analysis was performed after the initial candidate prioritization and served as a supplementary peptide-specific assessment rather than a criterion for candidate selection.

### 5.9. Molecular Docking

Molecular docking was performed to explore potential interactions between the candidate peptides and cancer-associated target proteins. The three-dimensional structures of the core target proteins were retrieved from the Protein Data Bank (PDB; https://www.rcsb.org/, accessed on 5 December 2025) and prepared using PyMOL 2.2.0 by removing water molecules, co-crystallized ligands, and non-standard residues. The peptide structures were initially generated using ChemDraw 15.1 and subjected to energy minimization using Chem3D 15.1 to produce initial three-dimensional conformations for docking input. AutoDock Tools 1.5.6 was used to prepare ligand and receptor files by adding polar hydrogens and assigning Gasteiger charges. The docking search space for each target protein was defined using a grid box of 126 × 126 × 126 points, with grid spacing and center coordinates determined individually for each target based on its three-dimensional structure. Molecular docking calculations were performed using AutoDock Vina, and results were visualized using PyMOL 2.2.0 and Discovery Studio 2019.

### 5.10. Cell Culture

A549 (human pulmonary epithelial cell) was purchased from National Collection of Authenticated Cell Cultures (Shanghai, China). The cells were cultured in RPMI-1640 medium supplemented with 10% fetal bovine serum in a humidified atmosphere containing 5% CO_2_ at 37 °C.

### 5.11. Cell Proliferation Assay

The A549 cells were plated in 96-well plates at a density of 1.0 × 10^4^ cells per well and incubated for 24 h. Following incubation, the cells were exposed to various peptide concentrations (0, 5, 15, 45, 135 μM) for 24, 48, and 72 h. Subsequently, 20 μL of MTT solution was added to each well. After an additional four-hour incubation, the supernatant was removed, and the resulting formazan product was dissolved in dimethylsulfoxide (DMSO). The absorbance at 490 nm was measured using a microplate reader. The cell proliferation rate was calculated using the formula: Proliferation rate = (OD treatment − OD blank)/(OD control − OD blank), where OD treatment represents the optical density of the treatment group, OD control represents the optical density of the control group, and OD blank represents the optical density of the empty well.

### 5.12. Statistical Analysis

Every experiment was repeated a minimum of three times. The data are presented as means with their respective standard deviations (SD). A one-way analysis of variance (one-way ANOVA) was employed to analyze the parameters. All statistical analyses were performed using GraphPad Prism Version 8.0.

## Figures and Tables

**Table 1:** The contents of protein in 14 batches of toad poison.

Sample Name	Content (mg/g)	Mean ± SD (mg/g)
TV-SD-1	252.99	263.71 ± 9.74
TV-SD-2	266.12	
TV-SD-3	272.01	
TV-SC-1	295.91	296.08 ± 1.44
TV-SC-2	294.73	
TV-SC-3	297.59	
TV-JS-1	293.55	290.86 ± 6.02
TV-JS-2	283.96	
TV-JS-3	295.07	
TV-DB-1	284.80	284.46 ± 2.20
TV-DB-2	286.48	
TV-DB-3	282.11	
TV-C-1	216.13	185.33 ± 43.56
TV-C-2	154.52	

**Table 2:** Score of 12 peptides with the probability of performing biological activities, according to the PeptideRanker server.

Rank	Peptide Sequence	PeptideRanker Scores ^1^
1	FGTQF	0.84
2	RPPGR	0.81
3	WEAWN	0.69
4	FSPDGH	0.68
5	NSQWG	0.63
6	FSTHL	0.62
7	AEWGR	0.57
8	NEYWR	0.56
9	RPFER	0.54
10	STWII	0.53
11	ACGVIGICQ	0.52
12	IAWTR	0.51

^1^ The score ranges from 0 to 1, and the closer to 1, the higher the probability of biological activity.

**Table 3:** Eighteen critical targets information of PPI network.

Name	Degree	Betweenness Centrality	Closeness Centrality
STAT3	68	0.10	0.77
MMP2	66	0.07	0.73
AKT1	66	0.07	0.76
JUN	64	0.03	0.73
MMP9	64	0.04	0.72
PTGS2	62	0.06	0.73
CCND1	62	0.03	0.70
CASP3	60	0.02	0.70
SRC	56	0.03	0.67
ICAM1	54	0.03	0.68
FOS	52	0.01	0.67
MMP3	48	0.03	0.64
CASP8	46	0.01	0.63
MMP1	44	0.01	0.62
CXCR4	44	0.01	0.63
CDK4	42	0.02	0.61
CDK2	40	0.01	0.60
HDAC1	40	0.01	0.59

**Table 4:** Specific information of the top 20 KEGG enriched pathways.

Pathways	Targets	Count	Fold Enrichment	−log10(*p* Value)
Pathways in cancer	ITGB1, JUN, GSTM1, MMP1, HDAC1, GSTP1, MMP2, STAT3, XIAP, CXCR4, FOS, PTGS2, MMP9, MAPK8, CASP8, CCND1, CDK4, CASP3, CDK2, AKT1, MAPK1, PPARG, BIRC3	23	8.32	15.09
TNF signaling pathway	JUN, MMP3, XIAP, FOS, PTGS2, MMP9, ICAM1, MMP14, MAPK8, CASP8, CASP3, AKT1, MAPK1, BIRC3	14	23.59	14.17
Kaposi sarcoma-associated herpesvirus infection	JUN, SRC, STAT3, HLA-A, FOS, PTGS2, ICAM1, MAPK8, CASP8, CCND1, CDK4, CASP3, AKT1, MAPK1	14	13.86	11.16
Lipid and atherosclerosis	JUN, MMP1, SRC, STAT3, MMP3, FOS, MMP9, ICAM1, MAPK8, CASP8, CASP3, AKT1, MAPK1, PPARG	14	12.51	10.59
IL-17 signaling pathway	JUN, MAPK8, MMP13, CASP8, MMP1, CASP3, MMP3, MAPK1, FOS, PTGS2, MMP9	11	22.48	10.59
Epstein–Barr virus infection	JUN, HDAC1, STAT3, HLA-A, ICAM1, MAPK8, CASP8, CCND1, CDK4, CASP3, CDK2, AKT1, HLA-DRB1	13	12.36	9.68
Human T-cell leukemia virus 1 infection	JUN, MMP7, XIAP, HLA-A, FOS, ICAM1, MAPK8, CCND1, CDK4, CDK2, AKT1, MAPK1, HLA-DRB1	13	11.25	9.20
Endocrine resistance	JUN, MAPK8, CCND1, SRC, CDK4, MMP2, MAPK1, AKT1, FOS, MMP9	10	19.60	8.96
AGE-RAGE signaling pathway in diabetic complications	JUN, MAPK8, CCND1, CDK4, CASP3, MMP2, STAT3, MAPK1, AKT1, ICAM1	10	19.21	8.88
Platinum drug resistance	GSTM1, CASP8, CASP3, GSTP1, MAPK1, XIAP, AKT1, BRCA1, BIRC3	9	23.68	8.61
Toxoplasmosis	ITGB1, MAPK8, CASP8, CASP3, STAT3, MAPK1, XIAP, AKT1, HLA-DRB1, BIRC3	10	17.31	8.48
Hepatitis B	JUN, MAPK8, CASP8, SRC, CASP3, STAT3, CDK2, MAPK1, AKT1, FOS, MMP9	11	13.04	8.24
Relaxin signaling pathway	JUN, MAPK8, MMP13, MMP1, SRC, MMP2, MAPK1, AKT1, FOS, MMP9	10	14.89	7.90
Small cell lung cancer	ITGB1, CCND1, CDK4, CASP3, CDK2, XIAP, AKT1, PTGS2, BIRC3	9	18.79	7.81
Measles	JUN, MAPK8, CASP8, CCND1, CDK4, CASP3, STAT3, CDK2, AKT1, FOS	10	13.92	7.64
Fluid shear stress and atherosclerosis	JUN, MAPK8, GSTM1, SRC, GSTP1, MMP2, AKT1, FOS, MMP9, ICAM1	10	13.82	7.61
Bladder cancer	CCND1, MMP1, SRC, CDK4, MMP2, MAPK1, MMP9	7	32.80	7.32
Viral carcinogenesis	JUN, CASP8, CCND1, HDAC1, SRC, CDK4, CASP3, STAT3, CDK2, MAPK1, HLA-A	11	10.36	7.28
Human cytomegalovirus infection	CASP8, CCND1, SRC, CDK4, CASP3, STAT3, MAPK1, CXCR4, AKT1, HLA-A, PTGS2	11	9.39	6.87
Apoptosis	JUN, MAPK8, CASP8, CASP3, MAPK1, XIAP, AKT1, FOS, BIRC3	9	12.71	6.47

**Table 5:** In silico ADMET-based screening of candidate peptides.

Peptide Sequence	HIA	PPB	BBB	CLplasma	logS	logP	Carcinogencity	A549 Cytotoxicity
FGTQF	Excellent	Excellent	Excellent	Excellent	−2.25	−0.29	Excellent	Excellent
RPPGR	Medium	Excellent	Excellent	Excellent	−2.27	−3.82	Medium	Excellent
WEAWN	Medium	Excellent	Excellent	Excellent	−3.05	−0.50	Excellent	Excellent
FSPDGH	Poor	Excellent	Excellent	Excellent	−2.41	−1.41	Excellent	Excellent
NSQWG	Excellent	Excellent	Excellent	Excellent	−2.98	−1.44	Excellent	Excellent
FSTHL	Poor	Excellent	Excellent	Excellent	−1.74	−0.12	Excellent	Excellent
AEWGR	Poor	Excellent	Excellent	Excellent	−3.78	−1.18	Excellent	Excellent
NEYWR	Poor	Excellent	Excellent	Excellent	−4.78	−1.26	Excellent	Excellent
RPFER	Poor	Excellent	Excellent	Excellent	−3.82	−1.59	Excellent	Excellent
STWII	Poor	Excellent	Excellent	Excellent	−2.61	0.76	Excellent	Excellent
ACGVIGICQ	Medium	Excellent	Excellent	Excellent	−2.37	0.84	Excellent	Excellent
IAWTR	Poor	Excellent	Excellent	Excellent	−2.76	0.27	Excellent	Excellent

**Table 6:** Criteria for the evaluation of in silico ADMET predictions.

Prediction Endpoint	Evaluation Criteria
Human Intestinal Absorption (HIA)	Excellent: 0–0.3 (Low HIA+ probability; high absorption) Medium: 0.3–0.7 (Moderate HIA+ probability) Poor: 0.7–1.0 (High HIA+ probability; low absorption)
Plasma Protein Binding (PPB)	Excellent: ≤90% (Low/Moderate binding; sufficient free drug) Poor: >90% (High binding; reduced free drug & low therapeutic index)
Blood–Brain Barrier Penetration (BBB)	Excellent: 0–0.3 (Low BBB+ probability; low penetration) Medium: 0.3–0.7 (Moderate BBB+ probability) Poor: 0.7–1.0 (High BBB+ probability; likely penetration)
Plasma Clearance (CLplasma)	Excellent: 0–5 mL/min/kg (Low clearance; prolonged exposure) Medium: 5–15 mL/min/kg (Moderate clearance) Poor: >15 mL/min/kg (High clearance; reduced exposure)
Aqueous Solubility (logS)	Acceptable logS range: −4 to 0.5
Lipophilicity (logP)	Acceptable logP range: 0 to 3
Carcinogenicity	Excellent: 0–0.3 (Low carcinogen probability; low risk) Medium: 0.3–0.7 (Moderate carcinogen probability) Poor: 0.7–1.0 (High carcinogen probability; high risk)
A549 Cytotoxicity (IC50)	Excellent: 0–0.3 (Low probability of A549 cytotoxicity) Medium: 0.3–0.7 (Moderate probability of A549 cytotoxicity) Poor: 0.7–1.0 (High probability of A549 cytotoxicity)

**Table 7:** Predicted pharmacokinetic and safety profiles of twelve candidate peptides assessed by pepADMET.

Peptide Sequence	Bioavailability (F)	BBB	HBN T1/2 (min)	Toxicity Probability	Cytotoxicity	LogD7.4
FGTQF	F−	BBB+	22.35	0.56	0.01	−1.00
RPPGR	F−	BBB+	0.35	0.05	0.00	−2.01
WEAWN	F−	BBB+	159.33	0.81	0.01	−0.51
FSPDGH	F−	BBB+	22.66	0.57	0.00	−1.78
NSQWG	F−	BBB+	251.02	0.34	0.00	−2.02
FSTHL	F−	BBB+	0.35	0.46	0.00	−1.22
AEWGR	F−	BBB+	37.90	0.75	0.02	−1.69
NEYWR	F−	BBB+	140.13	0.61	0.01	−1.54
RPFER	F−	BBB+	0.35	0.16	0.00	−1.72
STWII	F−	BBB+	86.90	0.21	0.00	−0.65
ACGVIGICQ	F−	BBB+	254.38	0.23	0.00	−0.32
IAWTR	F−	BBB+	81.17	0.38	0.00	−1.14

**Table 8:** Molecular docking parameters for the ten candidate target proteins.

Target	PDB ID	Resolution	Center Grid Box (x, y, z)	Grid Dimensions Points (x, y, z)	Spacing
STAT3	6NJS	2.70 Å	(−2.257, 19.470, 24.491)	126 × 126 × 126	1.000
MMP2	7XJO	2.00 Å	(42.206, −42.790, 9.428)	126 × 126 × 126	0.586
AKT1	3o96	2.70 Å	(6.290, −7.943, 17.261)	126 × 126 × 126	0.569
JUN	1JNM	2.20 Å	(31.631, 7.991, 10.901)	126 × 126 × 126	0.625
MMP9	1L6J	2.50 Å	(36.885, 38.845, 34.622)	126 × 126 × 126	0.692
PTGS2	5F19	2.04 Å	(22.594, 40.999, 39.56)	126 × 126 × 126	0.864
CCND1	2W96	2.30 Å	(5.936, 3.653, 59.281)	126 × 126 × 126	0.742
CASP3	1NME	1.60 Å	(27.820, 102.814,10.952)	126 × 126 × 126	0.558
SRC	1o4K	1.57 Å	(10.459, 19.241, 19.907)	126 × 126 × 126	0.419
ICAM1	1MQ8	3.30 Å	(9.916,7.543, −11.758)	126 × 126 × 126	0.953

**Table 9:** Sample information.

No.	Species	Sources (Geographical Location)
TV-SD-1	Toad poison	Shandong
TV-SD-2	Toad poison	Shandong
TV-SD-3	Toad poison	Shandong
TV-SC-1	Toad poison	Sichuan
TV-SC-2	Toad poison	Sichuan
TV-SC-3	Toad poison	Sichuan
TV-JS-1	Toad poison	Jiangsu
TV-JS-2	Toad poison	Jiangsu
TV-JS-3	Toad poison	Jiangsu
TV-DB-1	Toad poison	Dongbei
TV-DB-2	Toad poison	Dongbei
TV-DB-3	Toad poison	Dongbei
TV-C-1	Toad poison	National Institutes for Food and Drug Control
TV-C-2	Toad poison	National Institutes for Food and Drug Control

## Data Availability

The original contributions presented in this study are included in the article. Further inquiries can be directed to the corresponding authors.

## Abbreviations

The following abbreviations are used in this manuscript:

ADMET	Absorption, Distribution, Metabolism, Excretion and Toxicity
ACP	Anticancer Peptide
BBB	Blood–Brain Barrier
BP	Biological Process
CC	Cellular Component
DC	Degree Centrality
DTT	Dithiothreitol
FDR	False Discovery Rate
GO	Gene Ontology
HIA	Human Intestinal Absorption
IAA	Iodoacetamide
KEGG	Kyoto Encyclopedia of Genes and Genomes
MF	Molecular Function
MMP	Matrix Metalloproteinase
MTT	Thiazolyl Blue Tetrazolium Bromide
NCBI	National Center for Biotechnology Information
PDB	Protein Data Bank
PPB	Plasma Protein Binding
PPI	Protein–Protein Interaction
PTGS2	Prostaglandin-Endoperoxide Synthase 2
SD	Standard Deviation
STAT3	Signal Transducer and Activator of Transcription 3
TIC	Total Ion Current
